# Anti-Vascular Endothelial Growth Factor Antibody Suppresses ERK and NF-κB Activation in Ischemia-Reperfusion Lung Injury

**DOI:** 10.1371/journal.pone.0159922

**Published:** 2016-08-11

**Authors:** Chou-Chin Lan, Chung-Kan Peng, Shih-En Tang, Shu-Yu Wu, Kun-Lun Huang, Chin-Pyng Wu

**Affiliations:** 1 Division of Pulmonary Medicine, Buddhist Tzu Chi General Hospital, Buddhist Tzu Chi Medical Foundation, Taipei, Taiwan; 2 School of Medicine, Tzu-Chi University, Hualien, Taiwan; 3 Division of Pulmonary Medicine, Tri-Service General Hospital, Taipei, Taiwan; 4 Institute of Undersea and Hyperbaric Medicine, National Defense Medical Center, Taipei, Taiwan; 5 Department of Critical Care Medicine, Li-Shin Hospital, Tao-Yuan County, Taiwan; Leiden University Medical Center, NETHERLANDS

## Abstract

Ischemia-reperfusion (IR)-induced acute lung injury (ALI) is implicated in several clinical conditions like lung transplantation, acute pulmonary embolism after thrombolytic therapy, re-expansion of collapsed lung from pneumothorax or pleural effusion, cardiopulmonary bypass and etc. Because mortality remains high despite advanced medical care, prevention and treatment are important clinical issues for IR-induced ALI. Vascular endothelial growth factor (VEGF) has a controversial role in ALI. We therefore conducted this study to determine the effects of anti-VEGF antibody in IR-induced ALI. In the current study, the IR-induced ALI was conducted in a rat model of isolated-perfused lung in situ in the chest. The animals were divided into the control, control + preconditioning anti-VEGF antibody (bevacizumab, 5mg/kg), IR, IR + preconditioning anti-VEGF antibody (1mg/kg), IR+ preconditioning anti-VEGF antibody (5mg/kg) and IR+ post-IR anti-VEGF antibody (5mg/kg) group. There were eight adult male Sprague-Dawley rats in each group. The IR caused significant pulmonary micro-vascular hyper-permeability, pulmonary edema, neutrophilic infiltration in lung tissues, increased tumor necrosis factor-α, and total protein concentrations in bronchoalveolar lavage fluid. VEGF and extracellular signal-regulated kinase (ERK) were increased in IR-induced ALI. Administration of preconditioning anti-VEGF antibody significantly suppressed the VEGF and ERK expressions and attenuated the IR-induced lung injury. This study demonstrates the important role of VEGF in early IR-induced ALI. The beneficial effects of preconditioning anti-VEGF antibody in IR-induced ALI include the attenuation of lung injury, pro-inflammatory cytokines, and neutrophilic infiltration into the lung tissues.

## Introduction

Exposure of the lungs to periods of ischemia and the initiation of reperfusion causes ischemia-reperfusion (IR)-induced acute lung injury (ALI)[1], which is an important issue in lung transplantation. Lung transplantation provides a curative hope for many patients with end-stage pulmonary diseases. The shortage of donor organs remains a major limiting factor in the widespread application of lung transplantation [2]. Despite advances in organ preservation and peri-operative care, IR-induced ALI remains a significant cause of post-transplantation mortality and morbidity [2]. It is widely accepted that effective organ preservation is one of the keys to successful lung and heart-lung transplantation [2]. Although modern preservation techniques have revolutionized transplantation surgery, many investigators are still working toward a more reliable preservation method. IR-induced ALI sometimes occurs early after lung transplantation [3]. IR-induced ALI is one of the main causes of primary graft failure and contributes to early mortality after lung transplantation [2]. Therefore, there is an increasing interest in preservation of organs and to study early stage of IR-induced ALI.

The pathogenesis of IR-induced ALI is complicated and involves several biochemical, cellular, and molecular alterations [4, 5]. The pathologic process occurs when oxygen supply to the lungs has been compromised and then followed by a period of reperfusion. When reperfusion occurs, blood flow and oxygen are reintroduced to the ischemic lung parenchyma, facilitating a toxic environment through the creation of reactive oxygen species, the activation of the immune and coagulation systems, endothelial dysfunction, and apoptotic cell death [5].

The importance of the epithelial-endothelial barrier in IR-induced ALI is well established [6]. Pulmonary permeability is controlled by both endothelial and epithelial layers. IR-induced ALI causes widespread destruction on both sides of the epithelial-endothelial barrier and leads to hyper-permeability and pulmonary edema [7, 8]. At the onset of ALI, there is widespread destruction of the alveolar epithelial and endothelial membrane [7, 8], which leads to hyper-permeability and pulmonary edema [7, 8].

Vascular endothelial growth factor (VEGF), an angiogenic growth factor, is a member of a growing family of related proteins that include VEGF-A, -B, -C, -D and placental growth factor (PIGF)[9]. It is reported to have profound effects on endothelial cells, by regulating cell proliferation, apoptosis, and angiogenesis [9]. VEGF also plays important roles in maintaining alveolar epithelial cell survival [10]. Krebs and colleagues observed that VEGF either directly promoted epithelial regeneration or inhibited epithelial cell death [10]. Characterized as a vascular permeability factor, VEGF has also been implicated in the regulation of vascular permeability in many organ systems, including the lungs [11, 12] and can also result in the expression of inflammatory cytokines [13]. Since VEGF has role in regulating the epithelial-endothelial barrier, vascular permeability, and inflammatory cytokines, it may have roles in ALI.

The role of VEGF in ALI remains controversial as cited in many previous studies [14–17]. Some studies revealed that increased VEGF in the lungs is associated with lung injury [14–16]. Kaner et al. showed that the over-expression of VEGF through intra-tracheal administration of an adenoviral-mediated vector resulted in high-permeability pulmonary edema [14]. Kazi et al. found that increased expression of VEGF mRNA and protein in the lung was associated with IR and endotoxin-mediated ALI [15]. In mice exposed to lipopolysaccharides, increases in VEGF expression correlate with the development of inflammation, capillary leakage, and lung edema [16]. Such data indicates that elevated VEGF levels should be associated with pulmonary inflammation and edema.

However, other evidence points to the protective role of VEGF in ALI [9, 10, 17]. VEGF is known as a survival factor for endothelial cells and epithelial cells and inhibits apoptosis of these cells [9, 10]. Koh et al. showed that increased production of VEGF in the injured lung may contribute to the resolution of inflammation after lung injury [17]. According to these studies, VEGF is potentially protective in promoting repair of the alveolar-capillary membrane in ALI.

Since the association between VEGF and ALI remains controversial and there is only a paucity of studies regarding this issue. More studies about VEGF in ALI are necessary. It is important to study the role of VEGF in early stage of IR-induced ALI. This study therefore aimed to understand the role of VEGF in an IR-induced ALI rat model. Extracellular signal-regulated kinase (ERK) is one of the key mediators of signal transduction in response to VEGF [18]. As such, the roles of VEGF and downstream ERK in IR-induced ALI were investigated. Since VEGF is reported with pulmonary hyper-permeability and edema at the onset of ALI, it is hypothesized that VEGF elicits pulmonary edema and inflammation in IR-induced lung injury. Understanding the role of VEGF in IR-induced ALI processes is crucial for the management and helpful in developing new therapeutic strategies.

## Materials and Methods

### Isolation and perfusion of lungs

The National Science Council and the Animal Review Committee of the National Defense Medical Center (Taipei, Taiwan) approved the study protocol. The animals in this study were cared for in accordance with the ‘‘Guide for the Care and Use of Laboratory Animals” published by the United States’ National Institutes of Health.

Procedures regarding the preparation of isolated-perfused lung *in situ* in the chest were as previously described [19]. Briefly, male Sprague-Dawley rats were anesthetized through intra-peritoneal injection of pentobarbital sodium (50 mg/kg). After confirmation of deep anesthesia, tracheostomy was performed and a cannula was inserted into the trachea. The lungs were ventilated with a humidified gas mixture containing 5% CO_2_ in air at a frequency of 60 cycle/min, tidal volume of 3 mL, and end-expiratory pressure of 1 cm H_2_O. Median sternotomy was performed and heparin (1 U/g of body weight) was injected into the right ventricle.

A peristaltic pump (Model 1203, Harvard Apparatus) was used to perfuse the lungs with re-circulated perfusate composed of blood mixed with physiologic salt solution (119 mM NaCl, 4.7 mM KCl, 1.17 mM MgSO_4_, 22.6 mM NaHCO_3_, 1.18 mM KH_2_PO_4_, 1.6 mM CaCl_2_, 5.5 mM glucose, and 50 mM sucrose). Bovine albumin (4 g/dL) was added to maintain osmolarity of the perfusate. A cannula was placed in the left atrium through the left ventricle to collect the effluent perfusate for re-circulation.

The perfusion rate was kept at 8–10 mL/min by a roller pump and constant temperature (37°C) was maintained by a water bath. The preparation was placed on an electronic balance with the isolated lungs remaining *in situ*.

### Experimental protocols and induction of IR-induced ALI

The animals were divided into six groups (n = 8 per group): control (CTRL), control + preconditioning bevacizumab, 5 mg/kg (B5-CTRL), IR, IR + preconditioning bevacizumab, 1 mg/kg (B1-IR), IR + preconditioning bevacizumab, 5 mg/kg (B5-IR) and IR+ post-IR bevacizumab, 5 mg/kg (IR-B5). In the preconditioning groups (B1-IR and B5-IR), anti-VEGF antibody was administered before ischemia and in the IR-B5 group, anti-VEGF antibody was administered after IR.

The IR-induced ALI was performed with 30 minutes of ischemia by stopping ventilation and perfusion. After the 30-min ischemia, the lungs were re-perfused for 90 min and ventilated with 5% CO_2_ + 95% air. Lung weight and micro-vascular permeability (Kf) were measured at the baseline and post-IR.

All of the rats were further studied for lung histopathology, lung wet/dry weight ratio (W/D), total protein concentration, VEGF, ERK, tumor necrosis factor alpha (TNF-α), nuclear factor-kappa B (NF-κB), and inhibitor of NF-κB alpha (IκB-α).

### Microvascular permeability

An index of Kf was determined from the lung weight change induced by elevated pulmonary venous pressure (PVP). The measurement of Kf in isolated lungs was as previously described [19]. During ventilation and perfusion, the PVP was rapidly elevated by 10 cm H_2_O for 7 min. The slow, steady phase of weight gain as a function of time (ΔW/ΔT) was plotted on a semi-logarithmic paper and then extrapolated to zero time to obtain the initial rate of trans-capillary filtration. From this plot, Kf was defined as the y-intercept (gm/min) divided by PVP (10 cm H_2_O) and lung weight, and expressed in whole units of grams per minute per centimeter of H_2_O multiplied by 100 g [20].

### Total protein concentration and cytokine levels in bronchoalveolar lavage fluid (BALF)

At the end of the experiment, the lungs were lavaged twice with 2.5 ml isotonic saline. The returned fluid was collected by free drainage. The BALF was centrifuged at 200 x g for 10 min, and the total protein concentration in the supernatant was determined using bi-cinchoninic acid protein assay (Pierce, Rockford, IL, USA), while the TNF-α level was determined by commercially available enzyme-linked immuno-sorbent assay (ELISA) (R&D Systems Inc., Minneapolis, MN).

### Pulmonary edema

The lung W/D was used as an indicator of pulmonary edema. After the experiment, the right lung tissue was removed and the wet weight was determined to calculate for the lung weight. Part of the right middle lobe was weighed and then dried in an oven at 60°C for 48 hrs. The wet and dry weights were then measured to calculate the lung W/D.

### Lung histopathology

Histopathologic examination was performed to verify the micro-anatomic features of ALI imposed by IR and to assess the effects of anti-VEGF antibody. After all data were collected, the ventilation and perfusion were stopped and the lungs were removed from the isolated lung system. The lungs were fixed with 10% formaldehyde at 20 cmH_2_O infused through the trachea. The tissues were immersed in 10% formaldehyde fixative for 24 h, embedded in paraffin wax, and cut into 4–6 μm-thick sections using a microtome. The sections were then stained with hematoxylin and eosin (H&E) to assess interstitial edema and the degree of neutrophilic infiltration.

### Tissue neutrophil quantification

For tissue neutrophil quantification, H&E-stained sections were used to count the number of neutrophils per high power field (400 X) [21, 22]. To normalize the density of inflammation to the amount of lung tissue, neutrophil-to-epithelial cell (N/E) ratio was reported as indication of neutrophilic infiltration. For each slide, neutrophils and epithelial cells in the lung tissue were counted in 10 non-overlapping high power fields beginning at the periphery of the section. Each slide was examined by an observer blinded to the study [21, 22].

### Alveolar space area

For alveolar space area measurement, H&E-stained sections with high power field (400 X) were used. Lung images were captured by an NIKON E995 camera attached to an OLYMPUS BX50 microscope. The alveolar space area was determined on photographic prints and measured through point-counting digital planimeter (X-PLAN 380dlll, Ushikata Mfg, Japan). Thirty alveoli of each animal were measured randomly by a single researcher for all the variables studied. The mean alveolar space area was normalized to the control group.

### Enzyme-linked immunosorbent assay

The lung tissue VEGF was measured by separate sandwich ELISAs (R&D Systems, Minneapolis, MN, USA). Assays were performed in duplicate following the manufacturer's instructions. A specific monoclonal antibody was pre-coated onto a microplate. Standards and samples were pipetted into the wells. Subsequently, a polyclonal detection antibody was added. The resultant color develops was read spectrophotometrically in a plate reader. VEGF protein concentration in each sample was normalized to the total protein concentration (Bio-Rad, Hercules, CA) in the tissue homogenate.

### Immuno-blotting

Cytoplasmic and nuclear proteins were extracted from frozen lung tissue using the Nuclear/Cytosol Extraction kit (BioVision, Inc., Mountain View, CA) according to the manufacturer’s instructions. Protein concentrations were determined by BCA protein assay and equal amounts of lung homogenates (30μg/lane) were fractionated on 10–12% sodium dodecyl sulfate—polyacryl-amide gel electrophoresis (SDS-PAGE) gels and transferred to Hybond polyvinylidene fluoride membranes. The membranes were blocked by incubation inphosphate-buffered saline containing 0.1% Tween 20 and 5% non-fat milk for 1 h at room temperature.

The blots were incubated with antibodies of ERK, phosphorylated-NF-κB p65, and IκB-α (Cell Signaling Technology, Danvers, MA) overnight at 4°C. The blots were then washed three times for 10 min using phosphate-buffered saline containing 0.1% Tween 20. The blots were incubated with horseradish peroxidase linked anti-rabbit immunoglobulin G (1:40,000) or anti-goat immunoglobulin G (1:50,000) for 1 h at room temperature, and then washed three times in phosphate-buffered saline containing 0.1% Tween 20 for 10 min.

The bands were visualized using enhanced chemi-luminescence reagents and by exposing the blot to radiography film. The blots were then stripped and incubated with an anti-TATA antibody (for nuclear protein, diluted 1:1000; Abcam, Cambridge, MA) or anti-β-actin antibody (for cytoplasmic protein, diluted 1:10,000; Sigma, St. Louis, MO) to ensure equal loading.

### Data analysis

All statistical analyses were performed using the SPSS software 18.0 (SPSS Inc., Chicago, IL). All of the values were reported as means ± SD (standard deviation). Differences between groups were evaluated using Kruskal—Wallis followed by post hoc comparisons with Games Howell tests (intergroup comparison). Statistical significance was set at p<0.05.

## Results

### Preconditioning anti-VEGF antibody decreased IR-induced pulmonary edema and microvascular hyper-permeability (Fig 1)

**Fig 1:**
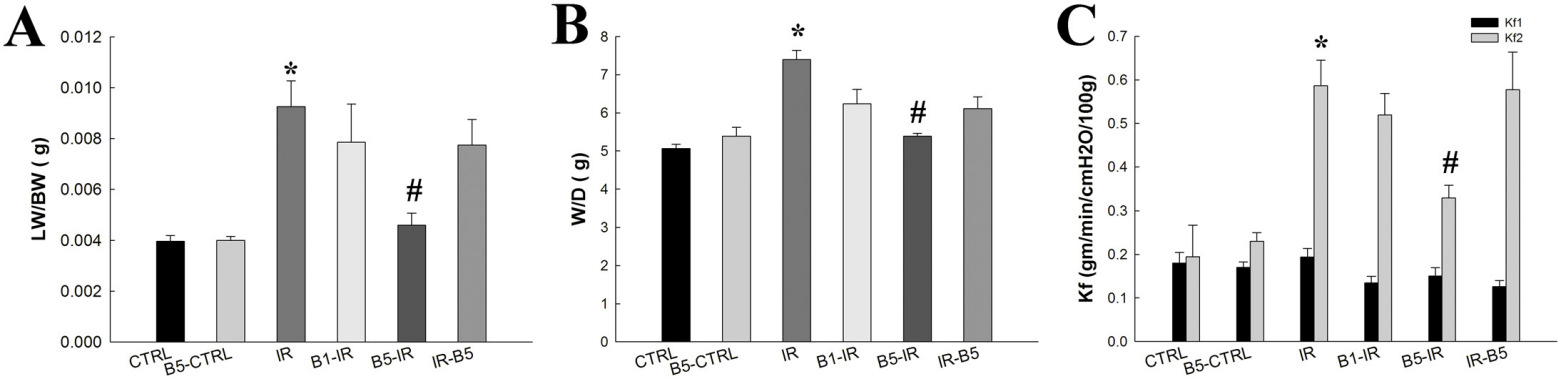
Preconditioning anti-VEGF antibody decreased IR-induced pulmonary edema and microvascular hyper-permeability. **(A)** Lung weight (LW) and wet/dry weight (W/D) was significantly increased in the IR group (p<0.05) and decreased in the B5-IR (p<0.05). **(C)** The Kf1 at baseline was similar in these groups (p>0.05) (Fig 1C). The Kf2 was significantly increased in the IR group and decreased in the B5-IR group (5 mg/kg) (p<0.05). There was a significant difference from the * CTRL (*p*<0.05) and ^#^IR (*p*<0.05) groups. Anti-VEGF antibody, anti-vascular endothelial growth factor antibody; IR, ischemia-reperfusion; ALI, acute lung injury. CTRL, control group; B5-CTRL, control + preconditioning bevacizumab, 5mg/kg group; IR, ischemia-reperfusion group; B1-IR, IR + preconditioning bevacizumab, 1mg/kg group; B5-IR, IR + preconditioning bevacizumab, 5mg/kg group; IR-B5, IR + post-IR bevacizumab, 5mg/kg group.

Lung weight (Fig 1A) and W/D (Fig 1B) was significantly increased in the IR group and significantly decreased in the B5-IR group (*p*<0.05).

The Kf1 at baseline was similar in these groups (*p*>0.05) (Fig 1C). The Kf2 was significantly increased in the IR group and significantly decreased in the B5-IR group (*p*<0.05).

### Preconditioning anti-VEGF antibody attenuated lung injury and neutrophilic infiltration (Fig 2)

**Fig 2:**
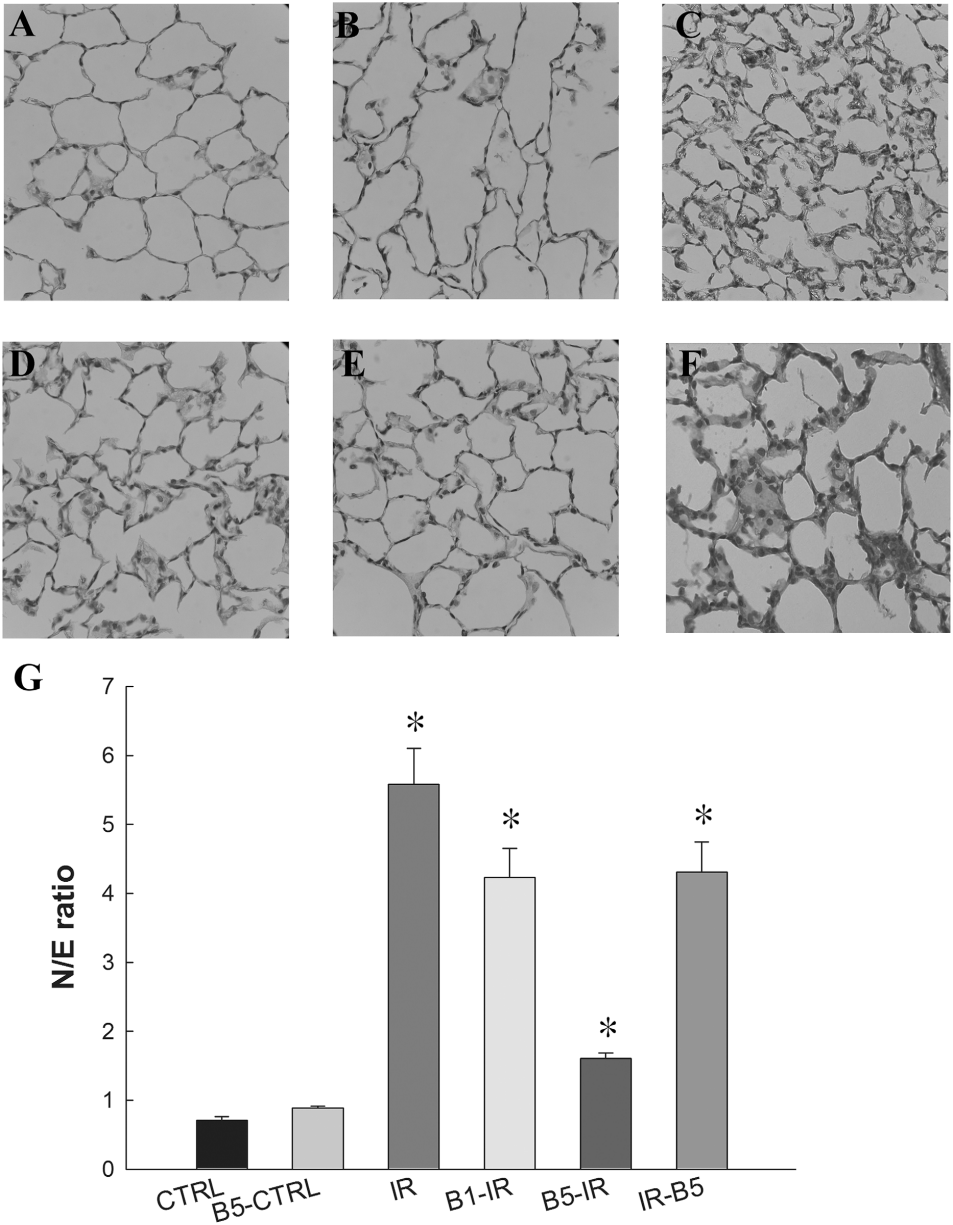
Preconditioning anti-VEGF antibody attenuated lung injury and neutrophilic infiltration. The CTRL **(A)** and B5-CTRL **(B)** groups had normal histology while the IR group **(C)** showed prominent neutrophilic infiltration and inter-alveolar septum thickening. Neutrophilic infiltration and inter-alveolar septum thickening was still prominent in B1-IR **(D)** but were markedly attenuated by preconditoning anti-VEGF antibody 5 mg/kg (B5-IR) **(E)**. The neutrophilic infiltration and inter-alveolar septum thickening were prominent in IR-B5 group **(F)**. **(G)** Neutrophilic infiltration was quantified and reported as N/E ratio. It was markedly increased in the IR group (p<0.05) and significantly decreased in B5-IR group (p<0.05 compared to the IR group). There was a significant difference from the *CTRL (*p*<0.05) and ^#^IR (*p*<0.05) groups. Anti-VEGF antibody, anti-vascular endothelial growth factor antibody; IR, ischemia-reperfusion; ALI, acute lung injury; N/E ratio: neutrophils to epithelial cells ratio. CTRL, control group; B5-CTRL, control + preconditioning bevacizumab, 5mg/kg group; IR, ischemia-reperfusion group; B1-IR, IR + preconditioning bevacizumab, 1mg/kg group; B5-IR, IR + preconditioning bevacizumab, 5mg/kg group; IR-B5, IR + post-IR bevacizumab, 5mg/kg group.

The CTRL (Fig 2A) and B5-CTRL (Fig 2B) groups had normal histology while the IR group showed prominent neutrophilic infiltration and inter-alveolar septum thickening (Fig 2C). Neutrophilic infiltration and inter-alveolar septum thickening was still prominent in B1-IR (Fig 2D) but were markedly attenuated by preconditoning anti-VEGF antibody 5 mg/kg (B5-IR) (Fig 2E). The neutrophilic infiltration and inter-alveolar septum thickening were still prominent in the IR-B5 group (Fig 2F).

Neutrophilic infiltration was quantified and reported as N/E ratio (Fig 2G). It was markedly increased in the IR group (*p*<0.05) and significantly reduced neutrophilic infiltration in the B5-IR group (*p*<0.05).

### Preconditioning anti-VEGF antibody attenuated IR-induced high expression of VEGF and ERK (Fig 3)

**Fig 3:**
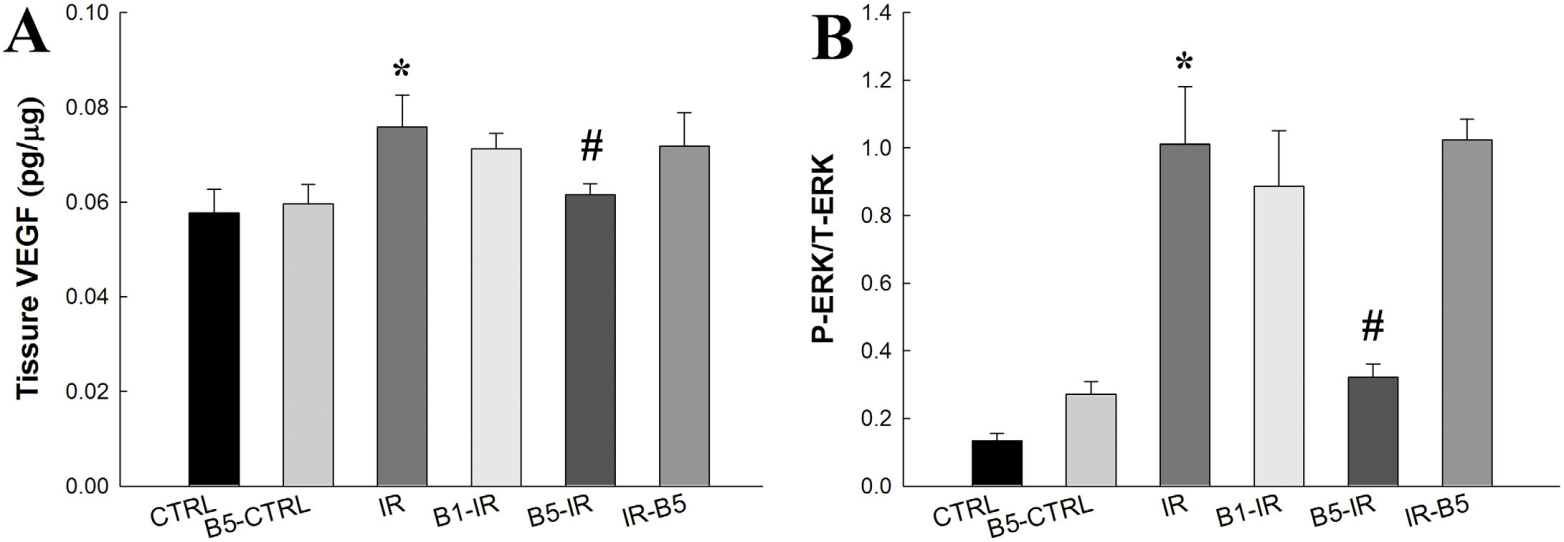
Preconditioning anti-VEGF antibody attenuated IR-induced high expression of VEGF and ERK. The levels of VEGF **(A)** and ERK **(B)** was significantly increased after IR (IR vs. control, *p*<0.05). However, the IR-induced expression of VEGF and ERK was attenuated in B5-IR (*p*<0.05). There was a significant difference from the *CTRL (*p*<0.05) and ^#^IR (*p*<0.05) groups. Anti-VEGF antibody, anti-vascular endothelial growth factor antibody; IR, ischemia-reperfusion; ALI, acute lung injury; ERK, extracellular signal-regulated kinase. CTRL, control group; B5-CTRL, control + preconditioning bevacizumab, 5mg/kg group; IR, ischemia-reperfusion group; B1-IR, IR + preconditioning bevacizumab, 1mg/kg group; B5-IR, IR + preconditioning bevacizumab, 5mg/kg group; IR-B5, IR + post-IR bevacizumab, 5mg/kg group.

Levels of VEGF (Fig 3A) and ERK (Fig 3B) were significantly increased in the IR group (IR vs. control, *p*<0.05). However, preconditioning anti-VEGF antibody (5 mg/kg) decreased the IR-induced expression of VEGF and ERK (*p*<0.05).

### Preconditioning anti-VEGF antibody decreased total protein concentration and pro-inflammatory cytokines in BALF (Fig 4)

**Fig 4:**
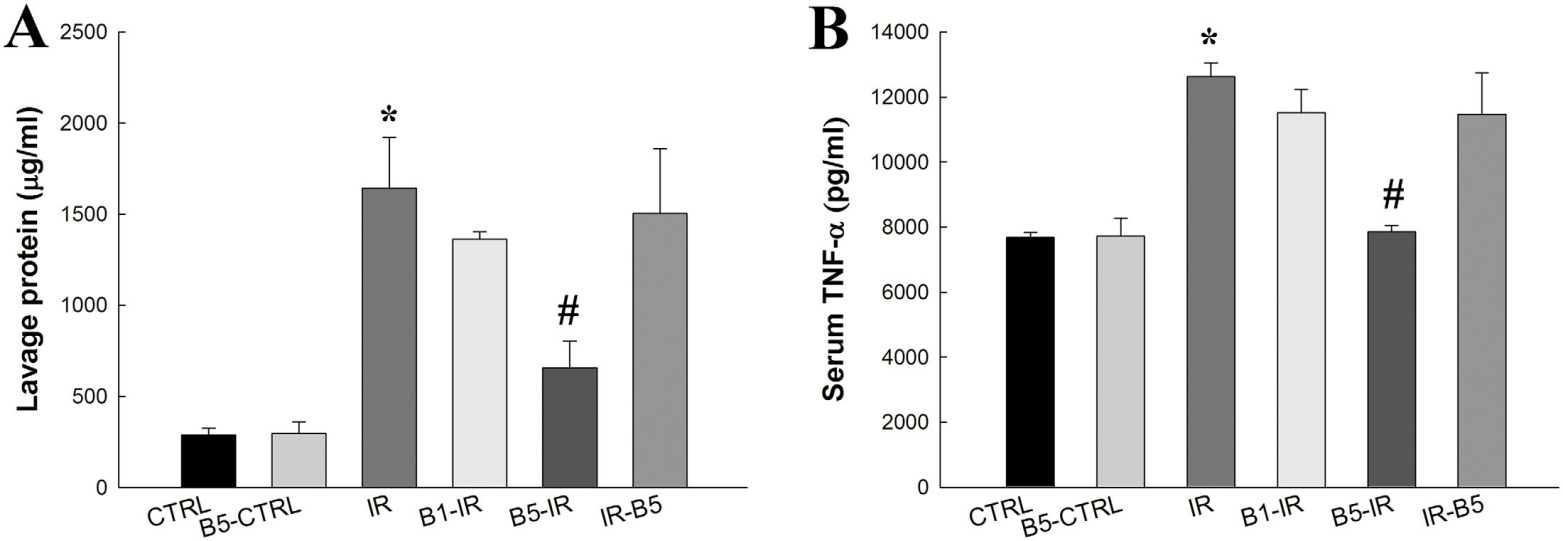
Preconditioning anti-VEGF antibody decreased total protein concentration and pro-inflammatory cytokines in BALF. **(A)** Total protein concentration and **(B)** TNF-α in BALF were significantly increased in IR (both *p*<0.05). Preconditioning anti-VEGF antibody (5 mg/kg) decreased IR-induced total protein concentration and TNF-α in BALF (both *p*<0.05). There was a significant difference from the * CTRL (*p*<0.05) and ^#^IR (*p*<0.05) groups. Anti-VEGF antibody, anti-vascular endothelial growth factor antibody; IR, ischemia-reperfusion; ALI, acute lung injury; BALF, broncho-alveolar lavage fluid; TNF-α, tumor necrosis factor-α. CTRL, control group; B5-CTRL, control + preconditioning bevacizumab, 5mg/kg group; IR, ischemia-reperfusion group; B1-IR, IR + preconditioning bevacizumab, 1mg/kg group; B5-IR, IR + preconditioning bevacizumab, 5mg/kg group; IR-B5, IR + post-IR bevacizumab, 5mg/kg group.

Total protein concentration (Fig 4A) and TNF-α (Fig 4B) in BALF were significantly increased in the IR group (both *p*<0.05). Preconditioning Anti-VEGF antibody (5 mg/kg) decreased IR-induced total protein concentration and TNF-α in BALF (both *p*<0.05).

### Preconditioning anti-VEGF antibody decreased IR-induced expression of NF-κB activation and nuclear translocation (Fig 5)

**Fig 5:**
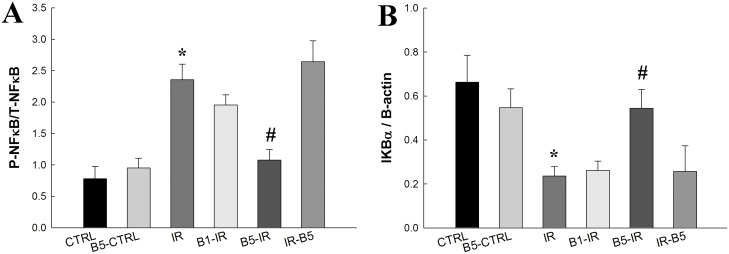
Preconditioning anti-VEGF antibody decreased IR-induced expression of NF-κB activation and nuclear translocation. After IR, the **(A)** cytoplasmic level of phosphorylated NF-κB p65 was increased, whereas **(B)** IκB-α was significantly suppressed (both *p*<0.05). Preconditioning anti-VEGF antibody group (5mg/kg) restored IκB-α and reduced phosphorylated NF-κB p65 levels (both *p*<0.05). There was a significant difference from the * CTRL (*p*<0.05) and ^#^IR (*p*<0.05) groups. Anti-VEGF antibody, anti-vascular endothelial growth factor antibody; IR, ischemia-reperfusion; ALI, acute lung injury; NF-κB, nuclear factor-kappa B; IκB-α, inhibitor of NF-κB alpha. CTRL, control group; B5-CTRL, control + preconditioning bevacizumab, 5mg/kg group; IR, ischemia-reperfusion group; B1-IR, IR + preconditioning bevacizumab, 1mg/kg group; B5-IR, IR + preconditioning bevacizumab, 5mg/kg group; IR-B5, IR + post-IR bevacizumab, 5mg/kg group.

In the IR group, the cytoplasmic level of phosphorylated NF-κB p65 (Fig 5A) was increased, whereas IκB-α (Fig 5B) was significantly suppressed (both *p*<0.05). Preconditioning anti-VEGF antibody (5 mg/kg) restored IκB-α and reduced phosphorylated NF-κB p65 levels (both *p*<0.05).

### Anti-VEGF antibody did not influence mean alveolar space area (Fig 6)

**Fig 6:**
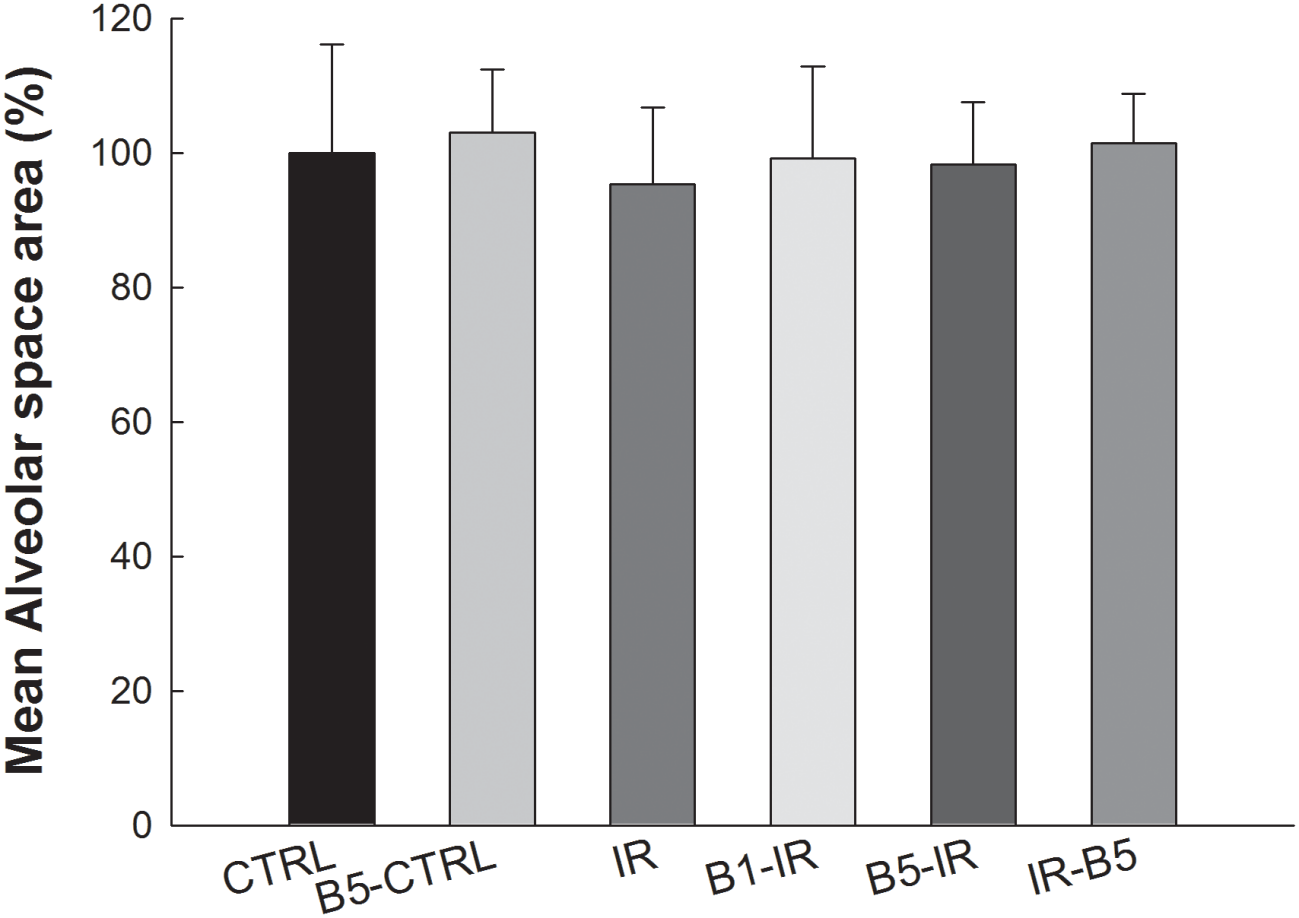
Anti-VEGF antibody did not influence mean alveolar space area. The mean alveolar space area was without significant differences between these six groups (all p>0.05). Anti-VEGF antibody, anti-vascular endothelial growth factor antibody; IR, ischemia-reperfusion; ALI, acute lung injury. CTRL, control group; B5-CTRL, control + preconditioning bevacizumab, 5mg/kg group; IR, ischemia-reperfusion group; B1-IR, IR + preconditioning bevacizumab, 1mg/kg group; B5-IR, IR + preconditioning bevacizumab, 5mg/kg group; IR-B5, IR + post-IR bevacizumab, 5mg/kg group.

The mean alveolar space area was without significant differences between these six groups (all *p*>0.05).

## Discussion

In the current study, there was increased VEGF in IR-induced ALI with concomitant increases in pulmonary permeability, pulmonary edema, neutrophilic infiltration, and pro-inflammatory cytokines. The downstream signals of VEGF, ERK, were also increased in IR-induced ALI. Preconditioning anti-VEGF antibody decreased VEGF and ERK, and attenuated IR-induced pulmonary hyper-permeability, neutrophilic infiltration, pulmonary edema, and pro-inflammatory cytokines. These results suggest that VEGF plays important roles in IR-induced ALI and the suppression of VEGF can attenuate IR-induced ALI.

The rapid increase in VEGF expression as a consequence of IR is via multiple mechanisms. Response to hypoxia and inflammation can result in the induction of VEGF [23]. Hypoxia-induced VEGF production is mediated by hypoxia-induced factor (HIF)-1α [24] by binding to a core sequence of the hypoxia-responsive element in the promoters of hypoxia-responsive genes, inducing the expression of VEGF [24]. After hypoxic exposure, VEGF expression increases in all regions of the lungs, but mostly in the alveolar epithelial cells [23]. Damaged pneumocytes in ALI also leads to increased release of VEGF. Zhang et al. also suggested that in the early stages of ALI, the acute inflammatory response induces VEGF release [25]. Alveolar macrophages and neutrophils represent a potential source of VEGF in ALI [26, 27], which stimulates the migration of both neutrophils and macrophages into the lungs. Both of these cell types produce VEGF after lung injury [26, 27], which is described in response to various inflammatory cytokines, such as TNF-α, IL-6, and IFN-γ [28]. Therefore, after lung injury, the recruited neutrophils and macrophages and increased pro-inflammatory cytokines lead to increased VEGF expression.

IR leads to increased VEGF expression, the increased VEGF further results in the aggravation of ALI. Previous studies suggest that VEGF functions primarily as pro-injurious molecules in the lungs [14, 29] as it is a potent factor in increasing the permeability of endothelial cells that leads to the passage of plasma components and leukocytes from blood vessel to tissues. The permeability-enhancing effects of VEGF underscore its significant role in acute inflammation to cause vascular leakage and the development of pulmonary edema [14, 29]. It is reported that VEGF affects endothelial morphology within 10 min, inducing the formation of fenestrae in the capillary sheet [30]. It may contribute to the development of pulmonary edema by altering the adherens junction complexes on the endothelium in the lungs [31].

Moreover, released VEGF induces the expression of cellular adhesion molecules, including E-selectin, intercellular adhesion molecule 1 (ICAM-1), and vascular cell adhesion molecule 1 (VCAM-1) in endothelial cells, and promotes the adhesion of leukocytes [9]. Activated leukocytes further cause injury through the release of toxic metabolites and protease [4, 32], while VEGF can lead to the expression of inflammatory cytokines [13]. Thus, lung injury leads to VEGF release, which subsequently leads to increased pulmonary vascular permeability and edema.

Several prior studies highlight the controversial roles of VEGF in ALI. Such observations are intriguing. Although VEGF can increase lung injury, there is also evidence to support its protective role in ALI. One possible explanation regarding this controversy is that VEGF may play different roles at different stages of ALI [25, 33].

Zhang et al. revealed the transient and overwhelming expression of VEGF in the early stage of ALI leads to the aggravation of lung injury with increased pulmonary permeability, edema and inflammation [25]. Mura et al. found that VEGF was significantly increased in BALF in the early stage of ALI [33]. The release of VEGF from alveolar epithelial cells and leukocytes induced by acute inflammatory response in the early stage of lung injury may increase the vascular permeability and contribute to the formation of interstitial edema in the lung [33]. Godzich et al. performed study about the role of VEGF in IR in rats and lung endothelial cells [34]. They suggested that activation of VEGF signaling is responsible for the increase in lung vascular permeability early after the onset of I/R in rats [34]. VEGF caused an increase in protein permeability across primary cultures of bovine macro- and microvascular lung endothelial cell monolayers [34]. Inhibition of VEGF-dependent cell signaling could prevent VEGF-mediated increase in protein permeability [34] Rapid release of VEGF in early I/R contributes to the development of pulmonary edema and inflammation [25, 33, 34]. In the current study, we suggested that preconditioning with anti-VEGF antibody attenuated the early release of VEGF and attenuated the IR-induced ALI.

However, during the late stage of ALI, VEGF may play protective role in recovery of lung injury [35]. VEGF is a potent angiogenic and endothelial survival factor in maintaining alveolar epithelial and endothelial cell survival [35]. This raises the consideration that improved lung injury in late stage could be explained by neovascularization in the lung and it take several days to develop neovascularization [14]. Medford and Millar suggested that VEGF has protective and regenerative effects on the pulmonary vascular endothelial and epithelial cells and facilitate repair after lung injury [36]. Increased VEGF in the latter stages of ALI may function to promote angiogenesis, an important component of lung repair whereas reduced VEGF and its receptors in alveolar epithelial cells due to tissue damage may lead to cell death [33]. Therefore, the early release of VEGF may aggravate lung injury [25, 33, 34], but VEGF in the late stage may have a role related to recovery [14, 33, 35, 36].

In addition, VEGF activates multiple signal transduction pathways, including extracellular signal-regulated kinases (ERK) [37]. The present study reveals increased EKR in IR-induced ALI. Studies about ERK in IR-induced lung injury are few and most are about ERK in IR injury in other organs [38–40]. Maddahi et al. have indicated that ERK plays a crucial role in IR-induced brain injury, inhibiting ERK mechanisms that can reduce infarct size [38]. The activation of the ERK pathway promotes the dissociation of NF-κB/IκB complexes, leading to NF-κB activation [39]. Singer et al. also reveal that ERK signaling results in the induction of IL-6, IL-8, and monocyte chemotactic protein-[40].

Maddahi et al. have also shown elevated microvascular expression of TNF-a, IL-1ß, and IL-6 following focal ischemia, and that this expression is transcriptionally regulated via the ERK pathway [38]. Breslin et al. have suggested that VEGF regulates endothelial hyper-permeability by signaling pathways involving ERK [37], whereas VEGF-induced hyper-permeability is inhibited by anti-sense DNA oligonucleotides directed against ERK [37]. There is also evidence suggesting that the activation of ERK contributes to cell death [37]. In the current study, we suggested that VEGF and ERK are involved in IR-induced ALI. Anti-VEGF antibody decreased VEGF and EKR after IR and attenuated lung injury.

A key transcription factor in cytokine gene expression, NF-κB is important in ALI [41]. In the current study, the NF-κB pathway and pro-inflammatory cytokines are activated after IR, as IR increases the phosphorylation of IKK-β, reduces IκB, and activates the NF-κB pathway. When cells are activated by inflammatory stimuli, IκB protein will be phosphorylated by IKK complex proteins. Phosphorylated IκB releases NF-κB dimers from the cytoplasmic NF-κB—IκB complex, allowing them to translocate to the nucleus. Then, NF-κB binds to κB enhancer elements of target genes to induce the transcription of pro-inflammatory genes [41].

In the classic pathway, the IKK-β sub-unit is mainly responsible for the phosphorylation of IκB-α and activation of NF-κB function [42]. This study reveals the concurrent activation of the NF-κB and VEGF pathways after IR. Anti-VEGF antibody decreases the NF-κB and VEGF pathways concurrently. In previous studies, NF-κB regulates the gene of VEGF [43], which is also known to induce activation of the NF-κB pathway in vascular endothelial cells [44]. In the present study, the concurrent activation of VEGF and NF-κB after IR was found. By administration of preconditioning anti-VEGF antibody, VEGF and NF-κB levels were decreased concurrently.

In the current study, preconditioning anti-VEGF antibody attenuated the IR-induced hyper-permeability and neutrophilic infiltration. However, the Kf2 and N/E ratio in the B5-IR group was still greater than the CTRL group. This result suggested that preconditioning anti-VEGF antibody can attenuate lung injury but not fully recover lung injury after IR. The pathogenesis of IR-induced ALI is complicated and involves several biochemical, cellular, and molecular alterations [4, 5]. The cellular mechanisms implicated in IR-induced ALI include innate and adaptive immunity, complement activation, activation of coagulation, activation of cell death pathways, and endothelial dysfunction [5]. The formation of reactive oxygen species, such as superoxide anions, hydrogen peroxide, and the most unstable and reactive, hydroxyl radicals, appears to play an important role in IR lung injury [5]. Preconditioning anti-VEGF antibody can attenuate but not fully recovery lung injury after IR for the complicated pathogenesis of IR-induced lung.

### Clinical implications

The lungs are one of the most vulnerable solid organs. There are several clinical conditions wherein IR-induced ALI is implicated [45–47]. Numerous attempts have been made to preserve lung viability after lung transplantation, pulmonary thrombo-embolectomy, or cardio-pulmonary by-pass [45–47]. IR-induced ALI is one of the main causes of primary graft failure contributing to mortality after lung transplantation [2] and sometimes occurs early after lung transplantation [3]. VEGF overexpression in early stage of IR leads to aggravation of lung injury. Therefore, it is important to study the role of VEGF in early stage of IR-induced ALI. The increasingly widespread use of VEGF antagonists in clinical conditions has focused even more attention on how these antagonists exert their effects. Understanding the relationship between VEGF and ALI may lead to the development of novel therapeutic interventions for this syndrome. To date, studies about anti-VEGF antibody in ALI are fairly limited. In the present study, preconditioning anti-VEGF antibody attenuated IR-induced ALI. This investigation provides impetus to consider approaches that address the role of anti-VEGF antibody in this clinical setting.

### Adverse effects of anti-VEGF antibody

The clinically reported adverse effects of anti-VEGF antibody, bevacizumab, are hemorrhage, arterial thromboembolic events, hypertension, proteinuria, anorexia, stomatitis, diarrhea and etc [48]. VEGF is an essential trophic factor required for the survival of lung epithelial and endothelial cells. Kasahara et al. suggested that chronic blockade of VEGF receptors could induce alveolar cell apoptosis and emphysema [49]. In the current study, we found that the mean alveolar space area was similar in the rats with or without anti-VEGF antibody. Kasahara performed chronic treatment of rats with the VEGF receptor blocker for three weeks and emphysema were found in the rats [49]. However, in the current study, we administered only short term anti-VEGF antibody and emphysema were not found.

### Limitations of study

In the current study, preconditioning anti-VEGF antibody before a period of ischemia reduces the IR-induced lung injury. This investigation provides a possible therapy in IR-induced lung injury. However, the present study has a number of limitations. The first is that this study is performed in the animal species and the isolated lung preparations used. There is still lack of human correlative data about anti-VEGF body in IR-induced ALI. Further studies in human beings in the clinical setting are warranted. Besides, the present study is limited to the early stage of ALI. Further investigation is required to address the long-term responses of anti-VEGF antibody in ALI.

## Conclusions

The rat isolated lung model of IR-induced ALI demonstrates increased expression of VEGF and ERK, and the concurrent micro-vascular hyper-permeability, pulmonary edema, increased inflammatory cytokines, and neutrophilic infiltration. Preconditioning anti-VEGF antibody is a protective strategy to pulmonary IR injury with attenuating pro-inflammatory cytokines, neutrophilic infiltration, pulmonary edema and micro-vascular hyper-permeability. This investigation provides impetus to consider approaches that address the role of anti-VEGF antibody in the clinical setting. However, further investigation is still required to address the responses of anti-VEGF antibody in ALI in human beings.

## Supporting Information

S1 File Data of lung weight, microvascular permeability. Lung weight/body weight and wet/dry weight was significantly increased in the IR group (p<0.05) and decreased in the B5-IR (p<0.05). The microvascular permeability was significantly increased in the IR group and decreased in the B5-IR group (5 mg/kg) (p<0.05).
(XLS)

S2 File Data of neutrophil count. Neutrophilic infiltration was quantified and reported as N/E ratio. It was markedly increased in the IR group (p<0.05) and significantly decreased in B5-IR group (p<0.05 compared to the IR group).
(XLS)

S3 File Data of VEGF and ERK. The levels of VEGF and ERK was significantly increased after IR (both *p*<0.05). However, the IR-induced expression of VEGF and ERK was attenuated in B5-IR (both *p*<0.05).
(XLS)

S4 File Data of total protein concentration, and pro-inflammatory cytokines. Total protein concentration and TNF-α in BALF were significantly increased in IR (both *p*<0.05). Preconditioning anti-VEGF antibody (5 mg/kg) decreased IR-induced total protein concentration and TNF-α in BALF (both *p*<0.05).
(XLS)

S5 File Data of NF-κB pathway. After IR, the cytoplasmic level of phosphorylated NF-κB p65 was increased, whereas IκB-α was significantly suppressed (both *p*<0.05). Preconditioning anti-VEGF antibody group (5mg/kg) restored IκB-α and reduced phosphorylated NF-κB p65 levels (both *p*<0.05).
(XLS)

S6 File Data of alveolar space area. The mean alveolar space area was without significant differences between these six groups (all p>0.05).
(XLS)
